# How Risky Is Mixed Martial Arts? Injury Rates and Patterns in Competitive Versus Recreational Athletes

**DOI:** 10.3390/healthcare14030409

**Published:** 2026-02-05

**Authors:** Lukas Groessing, Vasco Starke, Armin Runer, Friedemann Schneider, Markus Merkl, Wolfgang Zemann, Michael Schwaiger

**Affiliations:** 1 Department of Oral and Maxillofacial Surgery, Medical University Graz, Auenbruggerplatz 5, 8036 Graz, Austria; lukas.groessing@medunigraz.at (L.G.);; 2 Department for Orthopaedic Sports Medicine, Klinikum Rechts der Isar, Technical University of Munich, Ismaninger Str. 22, 81675 Munich, Germany; 3 Department of Orthopaedics and Traumatology, Medical University of Innsbruck, Anichstraße 35, 6020 Innsbruck, Austria

**Keywords:** sports injury, epidemiology, healthcare, economic impact, injury prevention, mixed martial arts, injury rate

## Abstract

**Background/Objectives**: Mixed martial arts (MMA) is a popular full-contact combat sport. The aim of this study was to determine injury mechanisms, rates, severity, patterns, circumstances, and the resulting economic healthcare consequences by conducting a detailed survey of competitive and recreational athletes. **Methods**: In 2023, MMA athletes were retrospectively questioned regarding their injuries in the last 24 months and the resulting healthcare impact (medical attention, hospitalization, incapacity to work). An injury was defined as any physical complaint resulting from MMA exposure. The severity of the injury was categorized according to the resulting restriction of sport participation (i.e., ‘severe’: more than four weeks of restriction). **Results**: A total of 112 participants (93% male; 41% non-competitive and 59% competitive) were included. All in all, 93,857 h of MMA activities were performed, and 127 injuries were recorded, resulting in an overall injury rate of 1.4 injuries per 1000 h of exposure. Non-competitive athletes reported significantly fewer total injuries in the past 24 months (95% CI 0.87–2.04; *p* = 0.003) and had significantly fewer severe or even critical injuries compared to competitive athletes (OR 0.55; 95% CI 0.21–1.43; *p* = 0.042). Head- and neck lesions (20%) were the most common injuries. The most common types of injury were joint sprains (21%) and ligament strains (17%). The healthcare burden of these sports-related injuries was minimal: By median, injuries led to zero days of hospitalization and incapacity to work, with no statistically significant differences between recreational and competitive athletes. **Conclusions**: Competitive athletes suffer more severe injuries compared to recreational athletes. Overall, injuries in MMA are rare, and the economic impact and burden on the healthcare system are negligible compared to other sport disciplines.

## 1. Introduction

Mixed martial arts (MMA) is a full-contact combat sport that has seen a rapid rise in popularity in recent decades and is frequently promoted as being highly violent and brutal [1,2,3].

MMA combines various types of martial arts, encompassing unarmed sports of Asian origin such as karate, judo and jiu-jitsu, along with more typical Western combat sports like boxing and wrestling, and their respective variations. The contests are conducted in a one-on-one format within a cage for a predetermined number of rounds.

It should be noted that the specific rules and regulations governing these contests may vary depending on the organization in question [2].

The athletes participating in this sport vary considerably in terms of their training experience, proficiency, age, technical capability, stamina, force output, weight, height, body composition, and their willingness to endure potential injuries [4].

The outcome of a contest is determined by one of the following: knockout, submission, technical knockout, decision, draw, disqualification, no decision, tap out, or choke [3].

Initially, the rule set was relatively simple: any technique was permitted except biting and eye-gouging, which resulted in a variety of injuries. In consequence, the Association of Boxing Commissions and Combative Sports introduced new rules in 2001. Notwithstanding the introduction of new rules, one of the primary objectives of a fight remains the rendering of one’s opponent unconscious [5].

It is common for fighters to sustain multiple injuries during a single bout. Prior research has indicated that the head, hand, and shoulder region are particularly vulnerable to injury [1,3,6].

Numerous studies have been conducted on the subject of injury rates and severity [1,4,6,7,8,9,10,11,12,13,14,15,16,17,18,19]. However, these studies have mostly focused on professional athletes. Consequently, there remains a significant research gap concerning athletes who participate in MMA recreationally and who do not compete professionally. This study was conducted to investigate the injury rate, severity, circumstances, return to sport (RTS), and economic impact (of injuries) among non-competitive athletes in comparison to competitive athletes.

## 2. Materials and Methods

### 2.1. Study Population

MMA athletes were invited worldwide. Participants were recruited using a multimodal approach via personal contacts with MMA associations, clubs, event organizers and social networks. Inclusion criteria were an age of over 18 years, regular MMA activity (training for a minimum of once per week) in the last 24 months, sufficient response to the questionnaire and consent to anonymous data processing. All athletes who did not fulfill these criteria were excluded. A comprehensive set of epidemiological data was collated, encompassing various demographic and athletic characteristics. Ethical approval was obtained from the local ethics committee (EK-Number: 35-316 ex 22/23; Medical University Graz; Approval date: 24 July 2024).

### 2.2. Data Acquisition

All data were collected through a self-assessment report using an anonymized online questionnaire between April 2023 and December 2023. The survey period covered the last 24 months retrospectively. Demographic and performance data on MMA were queried. In the case of an injury, questions were asked about the affected body region, the type, the severity, the cause, the circumstances of the injury, the protective equipment used, the resulting therapeutic measures and the economic consequences. The economic impact was calculated from the accident-related medical and dental care, the diagnostic imaging measures conducted (sonography, X-ray, MRI, CT), the subsequent therapeutic measures (surgery, conservative treatment, physiotherapy) and the resulting length of hospitalization and incapacity to work.

### 2.3. Injury Rate Calculation

The number of injuries per 1000 h of MMA exposure (training or competition) was used to calculate the injury rate to allow comparison with previous MMA studies [15,20]. Exposure, measured in hours, was defined as training and combat time. According to Fuller et al., an injury was defined as any physical complaint sustained by an athlete that results from practice or competition, irrespective of the need for medical attention or time loss from activities [21]. It is important to note that each injury occurrence may affect more than one body site and result in more than one injury. Injury severity was categorized by the resulting restriction from sport participation [17,22,23,24,25,26,27]. Injuries not resulting in training or competition limitations were classified as “slight”. Injuries that resulted in MMA limitations up to one week were classified as “mild”. Impairments of one to two weeks were classified as “moderate” and of two to four weeks as “serious”. A “severe” injury resulted in restrictions on participation in MMA for more than four weeks. An injury that resulted in permanent disability or death was defined as “catastrophic”.

### 2.4. Statistics

Statistical analysis was performed with SPSS Statistics (Version 27, IBM Corporation, Chicago, IL, USA). Prevalence tables were used to describe categorical variables. Continuous variables were reported as means and standard deviations. Chi-Square and Fisher’s Exact tests were used for categorical variables. Risk analyses were conducted using odds ratios (OR) and the 95% confidence interval (CI). The assessment of statistical significance between the parametric data was performed using Student’s *t*-test and the non-parametric data using the Mann–Whitney U Test. Level of significance was set at *p* < 0.05. The sample power was set at 0.8. The Shapiro–Wilk test was performed to assess normal distribution.

## 3. Results

### 3.1. Study Population

A total of 112 participants (104 men and eight women) were included. Details of the participants’ characteristics are provided in Table 1. The athletes mainly performed MMA in Austria (79.5%), followed by Germany (8.9%), while the rest were based internationally. Forty-six of the fighters were non-competitive athletes (41.1%) with an average experience of 2.7 years, and 66 were competitive athletes (58.9%) with a mean experience of 5.0 years. During the entire observation period, a total of 93,857 h of MMA exposure were observed.

### 3.2. Injury Rates, Patterns, and Circumstances

**Figure 1:**
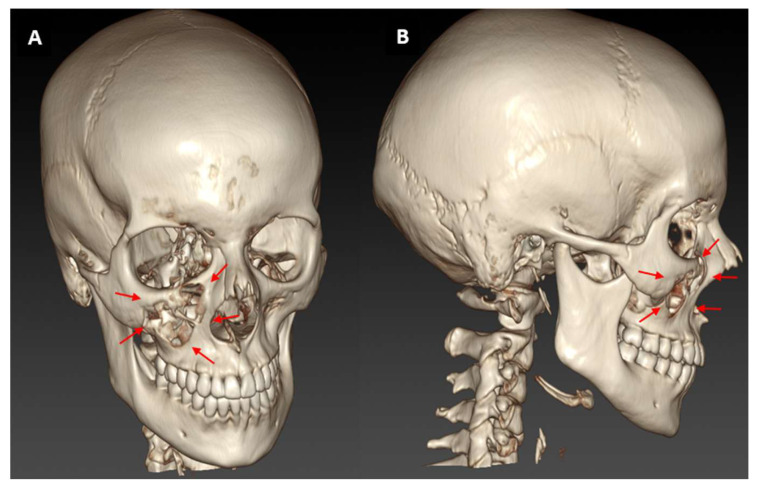
Maxillary fracture following MMA exposure: Frontal (**A**) and sagittal (**B**) views of 3D CT image of a 25-year-old female patient who presented to the Department of Oral and Maxillofacial Surgery after being impacted on the right zygomatic bone by an elbow during martial arts. The patient reported persistent dizziness, blurred vision in the left eye and headache, showing a fracture of the anterior wall of the right maxillary sinus with impaction of several lamellar bone fragments into the maxillary sinus and a non-displaced fracture of the lateral wall of the right maxillary sinus. In this case, the patient had no malocclusion, and the maxilla was fixed, which led to a conservative approach. Red arrows indicate the maxillary fracture on the right side.

A total of 127 injuries were sustained by 75 athletes during the study period, which corresponds to an injury rate of 1.4 injuries per 1000 h of exposure. Thirty-eight of the injured athletes (50.7%) reported one injury, 28 (37.3%) a second, three (4%) a third and six (8%) even a fourth injury. Non-competitive athletes were significantly less likely to injure themselves than competitive athletes (95% CI 0.87–2.04; *p* = 0.003). Thirty-three (26%) of the 127 injuries were classified as “severe”, 25 (19.7%) were “moderate”, 22 (17.3%) were classified as “slight”, 17 (13.4%) were “serious”, 15 (11.8%) were “mild”, and two (1.6%) were “catastrophic”. In 13 cases (10.2%), no information was provided on the severity of the injury. Non-competitive athletes had significantly fewer and less severe injuries than competitive athletes (OR 0.55; 95% CI 0.21–1.43; *p* = 0.042). The 127 injuries affected a total of 307 body sites. In 57 incidents, more than one body region was involved. The head and neck region was the most frequently affected localization (19.9%), followed by the ankle and foot region (15.3%). Figure 1 shows an exemplary case report of a midface fracture.

The most frequent types of injury were joint sprains (21.2%), followed by ligament strains (17.3%) and muscle strains (15.6%). Concussions accounted for 5.2% of all injuries. Table 2 and Table 3 provide further information. There were no significant differences between non-competitive and competitive fighters with regard to the type of injury and the body parts affected.

The majority of the athletes (95.5%) regularly used protection gear. Most frequently used were mouthguards (89.3%), MMA gloves (84.8), shin pads (77.7%), wrist bandages (59.8%), boxing gloves (56.3%), jock strap (52.7%), ankle bandages (18.8%), knee pads (17.9%), helmet (8%) and elbow pads (6.3%).

**Figure 2:**
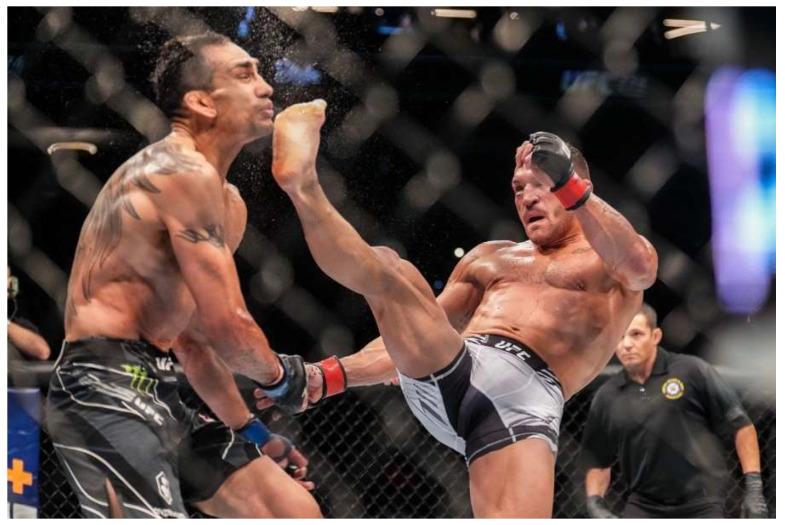
Kick to the head during an MMA fight.

In 33.1% of cases, the opponent was considered responsible for the injury, while in 26% of cases the injury was essentially self-inflicted. Most injuries (64.6%) occurred in a standing position, frequently as a result of a foot strike (25.2%). Figure 2 illustrates this vividly.

### 3.3. Economic Impact

44.9% of the injured athletes (*n* = 75) sought medical attention. While most cases could be treated conservatively (80.8%) primarily through physiotherapy (30.1%), surgery (13.7%) and dentistry (5.5%) were required in the remaining cases. In terms of diagnostic imaging, 53% of the injured athletes underwent X-rays, 24.1% magnetic resonance imaging, 15.7% ultrasound and 7.2% computed tomography. By median, the injury resulted in 0 days of hospitalization (SD for competitive athletes = 9.8 and non-competitive athletes = 2.3) and 0 days of incapacity to work (SD for competitive athletes = 10.9 and non-competitive athletes = 20.3). There were no significant differences between non-competitive and competitive fighters with regard to economic aspects.

## 4. Discussion

The primary finding of the present study is that the risk of injury in MMA for non-competitive athletes and competitive athletes is relatively low at 1.35 injuries per 1000 h of exposure in total. However, in instances of injury, 26.5% of these injuries were classified as severe, resulting in a duration of absence from sporting activities exceeding four weeks. The majority of these injuries occured during training and were typically caused by foot kicks. The most commonly injured regions were the head and neck, followed by the ankle/foot and hand/fingers. The present study revealed a statistically significant difference in injury rates between competitive athletes (1.03 injuries per 1000 h) and non-competitive athletes (1.56 injuries per 1000 h) with respect to MMA exposure.

Injury incidence was reported as injuries per 1000 h of exposure, reflecting a standardized approach widely adopted in sports injury epidemiology [23,24,25,26,28,29,30,31,32,33,34,35,36,37,38,39,40,41].

By contrast, much of the existing literature on combat sports has relied on combat-specific exposure measures, including injuries per bout or per fighting time, which limits direct comparability with injury data from other sports [3,7,8,9,10,11,13,14,15,16,20,42,43].

In other studies which take combat exposure into account, the injury rate reported ranges from 41 to 64.9 per 1000 combat minutes [15]. These rates for MMA are significantly higher than those observed in Olympic athletes per 1000 combat minutes, with the highest rates being observed in different combat sports, including boxing (9 per combat minute), judo (9.6 per combat minute), taekwondo (7.7 per combat minute) and wrestling (4.8 per combat minute) [42]. The relatively low number of injuries in this study can be attributed to the inclusion of training exposure in our analysis. In terms of the total number of injuries, competitive athletes have a higher frequency of injuries than non-competitive athletes (58.93%/41.07%). However, when considering injury rates per 1000 h of exposure, the rate for competitive athletes is lower, due to the remarkably high number of training hours they undertake.

Time off from sports is an important measure of injury severity. If injuries occurred, they were generally substantial. A third of non-competitive athletes and half of competitive athletes experienced injuries that resulted in more than two weeks’ absence from sport, which contrasts with another finding where only 10.4% of athletes needed more than 10 days to recover [22]. In comparison to other combat sports, wrestling (39.6%), followed by judo (35.9%), taekwondo (32.5%) and boxing (21%), had the highest time away from sports of more than seven days. This suggests that, in combat sports in which there is a focus on grappling instead of hand and foot strikes, ligament injuries, e.g., through various takedown techniques, occur more frequently and need more time for recovery. Prevention strategies should aim to target these types of injuries [44].

These results indicate that the overall frequency of injuries was relatively low. However, in instances where injuries did occur, they were predominantly substantial. This finding should be interpreted with caution, as the broad injury definition combined with a 24-month retrospective self-report design likely introduces recall bias. Minor or transient injuries that did not require medical attention or result in time loss may have been under-reported, whereas more severe injuries may have been more salient and therefore more likely to be recalled and reported by athletes. In addition, injuries sustained during occasional high-intensity or hard sparring rounds may be disproportionately remembered. Together, these factors may have contributed to an over-representation of severe injuries and an underestimation of the true overall injury frequency in this cohort.

The present study revealed that the head and neck were the most commonly affected body regions, a finding that is consistent with numerous other studies on MMA [1,8,17,20,22,45,46]. Nevertheless, the evidence from various studies is not entirely consistent with regard to the second most common location of injury. Some studies have identified the lower body as the site of injury most frequently affected, while others have highlighted the upper body as the most commonly injured region [8,13,20,22,45,46]. Two additional studies further subdivided the upper and lower body in their respective body regions. Regarding the upper body, the findings are comparable, indicating that the hand and fingers were the most affected, followed by the shoulder girdle and upper arm, as well as the elbow and forearms [22,46].

The regions of the lower extremity most frequently affected were the ankle and foot, the knee and calf, followed by the hip and thigh [22,46]. Injuries to the trunk and spine were the least common [17,22,46].

In terms of injury classification, this study presents a highly detailed and accurate classification system, in comparison to other studies that differentiate merely between fractures, lacerations, traumatic brain injury (TBI) and additional classifications [9,20].

In the present cohort, joint sprains were the most frequently observed injuries, followed by ligament and muscle strains. However, a systematic review and meta-analysis showed that lacerations were the most prevalent type of injury [1]. One hypothesis is that strains are more prevalent, and lacerations are less common, among MMA athletes who focus more on the wrestling and Brazilian Jiu-Jitsu aspects of the sport [47,48,49]. Soft tissue and joint injuries (including lacerations, abrasions, and contusions) account for 57.9% of the injuries in this study, aligning with other studies that report a combined average of 62% [14,15,50,51,52]. The current study reports a fracture incidence of 10% of injuries, while other reports indicate a range of 3.6 to 43.3% [3,9,12,15,20,22,51,52,53].

One of the most frequently sustained injuries in combat sports is a traumatic brain injury, which still seems to be vastly underestimated. The topic has been extensively researched and studied in numerous academic papers [9,54,55,56]. It is of the utmost importance to detect and manage these injuries effectively, as inadequate management can result in the development of persistent or chronic post-concussion syndrome [57]. The present study revealed that TBI was present in 5.2% of injuries (*n* = 16). This rate is considerably lower than that observed in a study of MMA, which reported an incidence of 62.3%, and is also lower than the prevalence reported in a study of UFC fighters, which was 45% [9,20].

The competitive athletes cohort sustained 68.24% of injuries during training, in comparison to 31.76% during competition. This is consistent with the findings of a previous study, which demonstrated a higher injury rate during training (77.9%) in comparison to injuries sustained in competition (22.1%) [22].

In regard to protective equipment, mouthguards serve to safeguard the teeth, gingival tissue, lips, and upper jaw by absorbing and redistributing energy [58]. The utilization of mouthguards has been demonstrated to be an efficacious method of preventing dental injuries [59]. In the cohort under examination, the mouthguard was the most frequently utilized protective equipment among the athletes, with a prevalence of dental injuries recorded at 2.9%. One study on MMA focused on dental injuries occurring in 0.1%, while other studies on martial arts have reported dental injuries in 10.47% of karate athletes and 3.7–35.9% of boxing athletes [12,60,61,62]. The prevalence of injuries in this study was insufficient to permit a meaningful comparison between the use and non-use of mouthguards.

Foot strikes and their devastating effect (Figure 1) lead to a high injury rate (25.2%). This is in contrast to a previous finding where fist strikes caused the highest number of injuries [9]. This could be due to the higher frequency of hand strikes and their almost exclusive use against the head of the opponent, whereas foot kicks deliver much more force [9]. The position in which most injuries occurred was standing, followed by lying with the back to the ground, which is consistent with other studies [9]. Given that the majority of fighting time is conducted in a standing position, this hypothesis seems plausible [63]. However, there are instances where competitors attempt to take their opponents to the ground in order to gain an advantageous position. This allows them to execute strikes with relative ease, although it is far harder for the opponent to evade them when in a grounded position. This is commonly referred to as “ground and pound” [64].

The vast majority of the surveyed athletes required no time off work and no hospitalization, but half of them saw a doctor and 5.5% saw a dentist. However, median values were used for calculation, which may mask the burden experienced by a subset of affected individuals. The frequency of doctor visits observed is significantly higher compared to a study from the Naval Medical Center San Diego, USA, where only 1 in 5 athletes sought medical attention [22]. An apparent explanation for this could be that healthcare in the countries primarily represented in this study is largely public, giving athletes low-threshold access to medical care [65].

To the best of our knowledge, there are no data on the annual costs of different sports to the healthcare system. Given the significant annual expenditure on sports injuries, the implementation of preventive strategies is crucial to reducing healthcare costs [66].

Other safety measures could include the use of structured injury prevention and post-injury RTS programs, and the adaptation of protective equipment (e.g., increased padding in gloves, the addition of shin and foot protectors, and the use of headgear). During training, athletes should prioritize controlled sparring and reduce the intensity of sparring activities. As other studies have indicated, it would be advisable to place an emphasis on the prevention of ligament injuries. Concerning prevention, the focus of measures should be on the improvement of protective equipment during training. Furthermore, changes to the rules of competition should be considered in order to minimize injuries to participants.

It is imperative to acknowledge the limitations inherent in this study. While both male and female athletes were represented, the study population was predominantly male. Consequently, the findings primarily reflect injury patterns in male MMA athletes, and caution should be exercised when generalizing these results to female MMA populations. The limited number of female participants restricts sex-specific analyses and limits the interpretability of individual female case examples within the broader context of the study. However, the majority of current studies exhibit a low proportion of female participation, with percentages ranging from as low as 5% to a maximum of 9% [4,12,22,46].

It is noteworthy that certain studies did not include any female participants [3,67].

The collection of data utilized self-report questionnaires, a method that is susceptible to recall bias. Furthermore, the study’s findings are exclusively based on acute injuries and do not encompass the athletes’ comprehensive injury history. Future studies should incorporate chronic and overuse injuries, and continuously survey over a longer period of time, in order to gain a comprehensive overview. Such studies should also evaluate individual injury risks, with particular reference to the long-term consequences of MMA exposure.

## 5. Conclusions

Competitive athletes appear to sustain injuries with greater frequency and severity than non-competitive participants.

While most athletes in the present cohort did not require time off work or hospitalization, a notable proportion still sought medical care and required clinical interventions, including surgical treatment. Overall, the findings support the safe practice of recreational MMA when suitable safeguards are implemented.

## Figures and Tables

**Table 1:** Participant characteristics.

Variable	Total	Competitive Athletes	Non-Competitive Athletes
Male	104 (92.9)	63 (95.5)	41 (89.1)
Female	8 (7.1)	3 (4.5)	5 (10.9)
Age [years] ^§^	25.5 ± 6.9	24.4 ± 6.5	27.1 ± 7.1
Height [cm]	179.6 ± 8.5	179.2 ± 7.7	180.1 ± 9.6
Weight [kg] ^§^	82.6 ± 16	81.3 ± 14.3	84.5 ± 18.1
BMI [kg/m2] ^§^	25.5 ± 4.1	25.9 ± 4.9	25.2 ± 3.5

BMI = body mass index; Data displayed as percent (number) if not otherwise stated; ^§^ data displayed as mean ± standard deviation.

**Table 2:** Body sites affected by injury.

Localization	Total		Non-Competitive Athletes		Competitive Athletes		OR [95%CI]	*p*-Value *
	N (%)	N/1000 h Exposure	N (%)	N/1000 h Exposure	N (%)	N/1000 h Exposure		
Head and neck	61 (19.8)	0.64	9 (13.2)	0.30	52 (21.7)	0.80	0.31 [0.12–0.77] *	0.007
Ankle and foot	47 (15.3)	0.50	10 (14.7)	0.33	37 (15.4)	0.57	0.51 [0.23–1.13]	0.086
Hand and fingers	43 (14.0)	0.45	14 (20.6)	0.47	29 (12.1)	0.45	0.91 [0.45–1.82]	0.748
Knee and calf	38 (12.3)	0.40	15 (22.1)	0.50	23 (9.6)	0.35	0.89 [0.40–2.01]	0.794
Shoulder girdle and upper arm	38 (12.3)	0.40	6 (8.8)	0.20	32 (13.3)	0.49	0.17 [0.05–0.59] *	0.002
Elbow and forearm	21 (6.8)	0.22	3 (4.4)	0.10	18 (7.5)	0.27	0.35 [0.10–1.25]	0.089
Trunk	19 (6.2)	0.20	2 (2.9)	0.06	17 (7.1)	0.26	0.23 [0.05–1.04]	0.038
Hip and thigh	18 (5.8)	0.19	4 (5.9)	0.13	14 (5.8)	0.21	0.51 [0.16–1.63]	0.245
Spine	16 (5.2)	0.17	2 (2.9)	0.06	14 (5.8)	0.21	0.20 [0.04–0.87] *	0.018
Other	7 (2.3)	0.07	3 (4.4)	0.10	4 (1.7)	0.06	1.44 [0.36–5.85]	0.923

Data displayed as percent (number) if not otherwise stated. * Mann–Whitney U test.

**Table 3:** Injury types.

Injury Types	N (%)	N/1000 h Exposure	Non-Competitive Athletes	N/1000 h Non-Competitive Athletes	Competitive Athletes	N/1000 h Competitive Athletes	*p*-Value *
			N (%)		N (%)		
Joint sprain	65 (21.2)	0.69	19 (48.7)	0.64	46 (61.3)	0.71	0.434
Ligament strain	53 (17.3)	0.6	15 (38.5)	0.51	38 (50.7)	0.59	0.155
Muscle strain	48 (15.6)	0.5	7 (17.9)	0.24	41 (54.7)	0.63	0.005
Fracture	31 (10.1)	0.3	4 (10.3)	0.14	27 (36.0)	0.41	0.004
Traumatic brain injury	16 (5.2)	0.2	3 (7.7)	0.1	13 (17.3)	0.20	0.074
Laceration	13 (4.2)	0.1	0 (0.0)	0	13 (17.3)	0.20	0.009
Contusion	9 (2.9)	0.1	1 (2.6)	0.03	8 (10.7)	0.12	0.058
Dental injuries	9 (2.9)	0.1	3 (7.7)	0.1	6 (8.0)	0.09	0.817
Organ injuries	3 (1)	0	0 (0.0)	0	3 (4.0)	0.04	0.404
Other/No sufficient information available	60 (19.5)	0.6	13 (33.3)	0.44	47 (62.7)	0.73	0.056

* Mann–Whitney U Test.

## Data Availability

The data presented in this study are available on request from the corresponding author. The data are not publicly available due to privacy and ethical restrictions.
